# Supra- and Infra-Renal Aortic Neck Diameter Increase after Endovascular Repair of a Ruptured Abdominal Aortic Aneurysm

**DOI:** 10.3390/jcm11051203

**Published:** 2022-02-23

**Authors:** Claire van der Riet, Richte C. L. Schuurmann, Angelos Karelis, Mehmet A. Suludere, Meike J. van Harten, Björn Sonesson, Nuno V. Dias, Jean-Paul P. M. de Vries, Martijn L. Dijkstra

**Affiliations:** 1 Division of Vascular Surgery, Department of Surgery, University Medical Center Groningen, BA60, Room W4.242, P.O. Box 30.001, 9700 RB Groningen, The Netherlands; r.c.l.schuurmann@umcg.nl (R.C.L.S.); m.a.suludere@umcg.nl (M.A.S.); m.j.van.harten@umcg.nl (M.J.v.H.); j.p.p.m.de.vries@umcg.nl (J.-P.P.M.d.V.); m.l.dijkstra@umcg.nl (M.L.D.); 2 Department of Thoracic Surgery and Vascular Diseases, Vascular Centre, Skåne University Hospital, 21428 Malmö, Sweden; karelisangelos@gmail.com (A.K.); bjorn.sonesson@med.lu.se (B.S.); nuno.dias@med.lu.se (N.V.D.); 3 Department of Clinical Sciences Malmö, Lund University, 22100 Lund, Sweden

**Keywords:** ruptured aneurysm, abdominal aortic aneurysm, hypovolemic shock, hypotension, endovascular procedures

## Abstract

Hypovolemia-induced hypotension may lead to an aortic diameter decrease in patients with a ruptured abdominal aortic aneurysm (rAAA). This study investigates the changes in supra- and infra-renal aortic neck diameters before and after endovascular aortic aneurysm repair (EVAR) for rAAA and the possible association with endograft apposition. A retrospective cohort study was conducted including 74 patients treated between 2010 and 2019 in two large European vascular centers. Outer-to-outer wall diameters were measured at +40, +10, 0, −10, and −20 mm relative to the lowest renal artery baseline on the last pre- and first post-EVAR computed tomography angiography (CTA) scan in a vascular workstation. Endograft apposition was determined on the first post-EVAR CTA scan. The post-operative diameter was significantly (*p* < 0.001) larger than the preoperative diameter at all aortic levels. The aortic diameter at +40 mm (supra-renal) and −10 mm (infra-renal) increased by 6.2 ± 7.3% and 12.6 ± 9.8%, respectively. The aortic diameter at +40 mm increased significantly more in patients with low preoperative systolic blood pressure (<90 mmHg; *p* = 0.005). A shorter apposition length was associated with a higher aortic diameter increase (R = −0.255; *p* = 0.032). Hypovolemic-induced hypotension results in a significant decrease in the aortic diameter in patients with an rAAA, which should be taken into account when oversizing the endograft.

## 1. Introduction

A ruptured abdominal aortic aneurysm (rAAA) is a life-threatening condition with a 30-day overall mortality rate of 65–85% [1]. The use of endovascular aneurysm repair (EVAR) for rAAA has increased in recent decades and is now the preferred treatment in many countries [2]. Endovascular treatment of an rAAA can be challenging because of the limited amount of time to plan the procedure and to size the endograft, often combined with hostile aorto-iliac anatomy [3]. The planning includes accurate measurement of the aortic neck anatomy in curved three-dimensional vascular reconstructions to define if the anatomy is suitable for elective repair [4]. The required measurements are stated in the indications for use of the used endograft, and include length, diameter, and angulation of the aortic neck. 

The endograft diameter should exceed the diameter of the aortic neck to achieve radial force and an effective seal. An oversizing of 10% to 25% has been suggested to be sufficient in elective EVAR. In emergency EVAR, however, an oversizing of 30% is advised to account for the hemodynamic condition of the patient [5,6]. It is, however, unknown how much the juxta-renal aortic diameter is influenced by hypovolemia in patients with an rAAA. Previous studies have shown that the aortic diameter increases after open repair in rAAA patients, independent of the preoperative aortic diameter [7]. 

Sufficient circumferential apposition of the endograft within the aortic wall is essential for sustainable EVAR outcomes. The shortest apposition length is, however, not assessed on standard post-operative computed tomography angiography (CTA) scans. 

The first aim of this study is to evaluate the aortic diameters before and after EVAR at levels proximal to the endograft and at the level of the endograft. The second aim is to correlate the diameter increase above the endograft with the preoperative systolic blood pressure. The hypothesis is that hypovolemic-induced hypotension results in a significant decrease in the aortic diameter. The third aim is to assess the length of circumferential apposition of the endograft in the infra-renal neck and to compare this to the preoperative neck length.

## 2. Materials and Methods

This retrospective multicenter study was conducted in accordance with the Strengthening the Reporting of Observational Studies in Epidemiology (STROBE) guidelines and was performed in line with the Declaration of Helsinki. The institutional review board approved the study (Ref No. 201900392). The requirement for informed consent was waived according to institutional policy on retrospective research. 

### 2.1. Patient Selection 

The study used a retrospective database of patients with rAAA who underwent emergency surgery for an infra-renal AAA between 2010 and 2019 in two European tertiary vascular referral centers. Inclusion criteria were CTA-confirmed rupture of the AAA and endovascular treatment with a standard infra-renal bifurcated endograft. An rAAA was defined as a visible retroperitoneal hematoma on CTA confirmed by an experienced radiologist. Exclusion criteria were a symptomatic AAA without rupture, missing post-operative CTA within 90 days of the procedure, insufficient quality of the pre- or post-EVAR CTA scan, or additional proximal fixation (e.g., endoanchors, additional cuffs, or bare-metal stents for proximal reinforcement, leading to scattering and inaccurate measurements).

### 2.2. CTA Scan Protocol

Images were acquired on a Siemens Definition EDGE or a Definition Flash CT scanner (Siemens Healthcare GmbH, Erlangen, Germany), with the standard local hospital CTA protocol. Scan parameters of the University Medical Center Groningen were variable tube voltage according to the Care-kV protocol, or variable tube current according to the Care Dose 4D protocol, 0.8 mm pitch, 128 × 0.6 mm collimation, and 0.5 s rotation time. Per CTA scan, 100 mL (4 mL per second) of diluted contrast (Xenetrix 300; Guerbet, Sulzbach, Germany) was administered intravenously. Acquisition was obtained in the early arterial phase. Scan parameters of the Skåne University hospital were tube voltage 80/140 kV, 0.55 mm pitch, 128 × 0.6 mm collimation, and 0.55 s rotation time. Acquisition was obtained in the arterial phase after intravenous injection of an individual contrast volume based on body weight (Omnipaque 350 mg/mL; GE Healthcare, Oslo, Norway; maximum dose weight of 80 kg and dose of 300 mg/kg). Bolus tracking was used with the region of interest (ROI) placed at the level of the diaphragm with a threshold of 120 Hounsfield units (HUs).

### 2.3. Data Collection and CTA Measurements

Patient characteristics, clinical details, and CTA measurements were collected in REDCap 10.0.23 (Vanderbilt University, Nashville, TN, USA). The clinical details, documented preoperatively at the emergency department and postoperatively at the intensive care unit or surgical ward, were retrospectively collected from the electronic patient records. Hypovolemic-induced hypotension was defined as preoperative systolic blood pressure of <90 mmHg according to the shock criteria [8]. The preoperative systolic blood pressure was measured routinely with an arterial line. 

**Figure 1:**
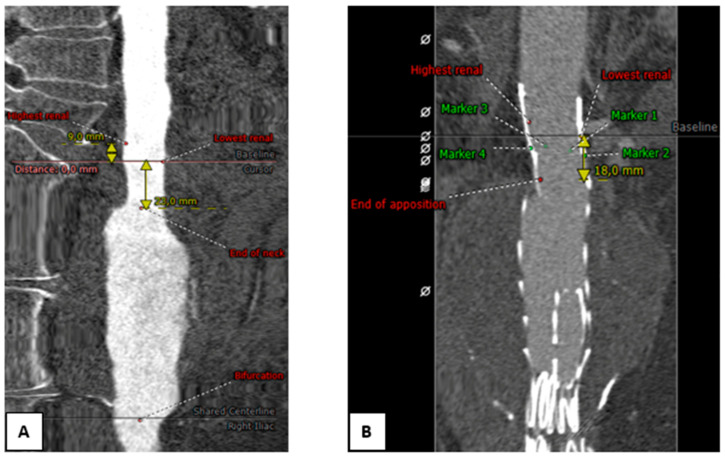
Measurements in 3mensio vascular workstation on the preoperative computed tomography angiography (CTA) scan. (**A**) Baseline is set to the distal edge of the lowest renal artery. The end of the aortic neck is defined as the first slice that exceeds a 10% diameter increase compared with baseline; (**B**) positioning of three-dimensional coordinate markers. CTA coordinates are obtained from the lowest and highest renal arteries (red), the end of circumferential apposition (red), and of four radiopaque markers that define the proximal edge of the endograft fabric (green).

The preoperative and first postoperative CTA scans were assessed in a 3mensio 10.2 vascular workstation (Pie Medical Imaging BV, Maastricht, the Netherlands) following a predefined measurement protocol (Figure 1) [9]. The measurements were performed by one experienced observer (M.S.), to avoid inter-observer variability. Measurements were randomly verified and outliers were checked for correctness by a second observer (R.S.). 

A center lumen line was semi-automatically created from the celiac trunk to the aortic bifurcation. The baseline was set at the inferior border of the orifice of the lowest renal artery. In case of an accessory renal artery which was not over-stented, this accessory renal artery was used as baseline. The distal end of the aortic neck on the preoperative CTA scans was defined as the first slice with >10% diameter increase compared with baseline [10]. The neck length was measured over the centerline between the renal artery baseline and the distal end of the neck. On the preoperative CTA scan, three-dimensional (3D) coordinate markers were placed at the inferior border of the orifice of the left and right renal artery and at the end of the aortic neck. On the post-operative CTA scan, 3D coordinate markers were placed at the renal artery orifices, at the radiopaque markers that are located 1–2 mm below the proximal edge of the endograft fabric, and at the first slice where circumferential apposition between endograft and aortic wall was lost (Figure 1B). Localized gutters or infolding of the top of the fabric was not determined in any of the patients. 

The aortic diameter was measured at five different levels relative to the renal artery baseline (+40, +10, 0, −10, and −20 mm) as the average from the largest diameter and its orthogonal diameter, measured from adventitia to adventitia. The +40 mm level is above the bare stent of the Zenith and Endurant endografts, +10 mm is at the height of the bare stent, 0 mm (baseline) is at the inferior border of the orifice of the lowest renal artery, −10 mm is at the level of the aortic neck that is covered by the endograft fabric in most patients, and −20 mm is at the end of the aortic neck in most patients. The endograft main body nominal diameter was obtained from the patient records. There were two endograft oversizing parameters defined: the planned pre-EVAR endograft oversizing and the effective post-EVAR endograft oversizing [11]. The planned pre- and the effective post-EVAR endograft oversizing were calculated from the nominal endograft diameter and the pre- and post-EVAR neck diameter at the lowest renal artery baseline, respectively, as shown in Formulas (1) and (2):

(1) $$\left(\frac{nominal\;endograft\;diameter}{preoperative\;neck\;diameter}-1\right)\times \;100\%$$

(2) $$\left(\frac{nominal\;endograft\;diameter}{postoperative\;neck\;diameter}-1\right)\times \;100\%$$

The shortest apposition length was calculated using vascular image analysis (VIA) prototype software (Endovascular Diagnostics BV, Utrecht, The Netherlands), according to previously published methods [12]. The coordinates of the renal arteries, proximal endograft fabric, and the distal end of circumferential apposition, along with the centerline and a segmentation of the aortic lumen, were exported to VIA. The software automatically calculated the circumferential surface area of apposition of the endograft with the aortic wall. The shortest apposition length is the shortest distance between the circumference of the proximal endograft fabric and the first slice where circumferential apposition of the endograft with the aortic wall was lost. The ratio of the preoperative neck length that is used postoperatively for apposition of the endograft was calculated. A ratio of 1 equals a neck length which was completely utilized, whereas a ratio of <1 means that less than the anticipated neck length is effectively used for the seal.

### 2.4. Statistics

Histograms, quantile–quantile plots, and the Kolmogorov–Smirnov test were assessed to verify normal distribution of the data. Preoperative neck length, postoperative shortest apposition length, the shortest apposition length/neck length ratio, planned pre-EVAR oversizing, and effective post-EVAR oversizing were not normally distributed. These variables are presented as the median (interquartile ranges). The other variables were normally distributed and are presented as the mean ± standard deviation (SD). Categorical outcomes are presented as the frequency and percentage. 

Significant differences between pre- and postoperative diameters were tested with the paired *t*-test, and significant differences between the oversizing were tested with the nonparametric Wilcoxon signed-rank test. Correlations between the increase in aortic diameter, the preoperative aortic diameter, and the intended oversizing with the systolic blood pressure were tested with the Pearson correlation coefficient (R). Correlations between the increase in infra-renal aortic diameter and the shortest apposition length were tested with the Spearman correlation (ρ). The independent samples *t*-test was used to compare the supra-renal diameter change between patients with and without preoperative hypovolemic-induced hypotension (systolic blood pressure of <90 mmHg). Statistical analysis was conducted using SPSS 27 software (IBM Corp, Armonk, New York, NY, USA). A probability (*p*) value of <0.05 was considered indicative of statistical significance.

## 3. Results

In total, 113 patients from the Skåne University Hospital and 60 patients from the University Medical Center Groningen met the inclusion criteria. Of these 173 patients, 99 patients were excluded from the analysis due to a symptomatic AAA without rupture (n-91); missing post-operative CTA within 90 days of the procedure (n-3); insufficient quality of the pre- or post-EVAR CTA scan (due to insufficient contrast agent opacification of the aorta for adequate measurements; n-2); or additional proximal fixation techniques, such as with EndoAnchors, additional cuffs, or bare-metal stents for proximal reinforcement, leading to scattering and inaccurate measurements (n-3).

The baseline characteristics of the 74 included patients are reported in Table 1, and their hemodynamic and renal parameters are shown in Table 2. The pre- and postoperative systolic blood pressure was available for 63 patients, and was significantly lower pre-EVAR compared to post-EVAR (*p* < 0.001), and 23 of these patients (33%) were in hypovolemic shock. Pain was reported by 61 patients and 1 patient was resuscitated during the pre-EVAR period. Implanted endografts were Cook Zenith (72%), Gore Excluder (21%), and Medtronic Endurant (7%). The median procedure time was 145 (119, 180) minutes, and the median blood loss was 200 (100, 625) mL. The median time elapsed between the EVAR procedure and the first post-EVAR CTA scan was 36 (19, 47) days. A type IA endoleak was detected on the first postoperative CTA scan in three patients, two of whom underwent a reintervention.

**Figure 2:**
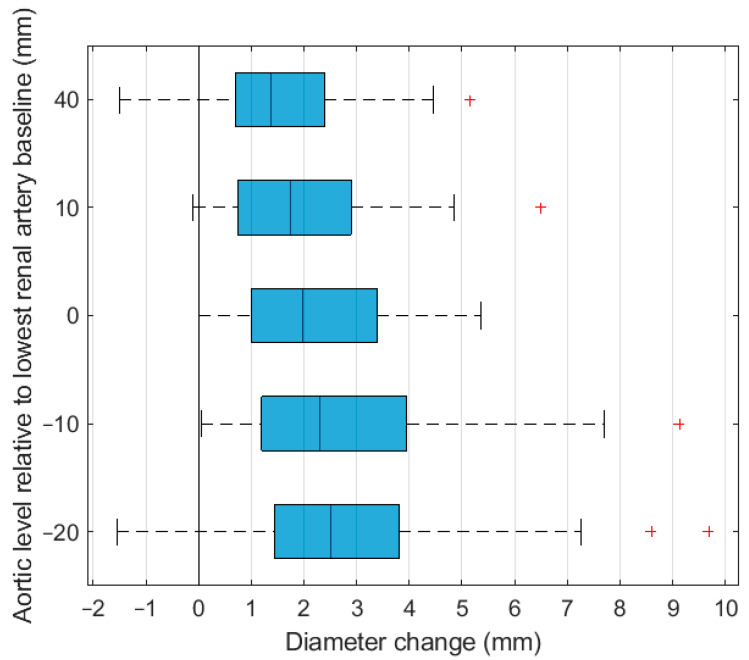
Diameter change at five aortic levels on the first computed tomography angiography scan after endovascular aneurysm repair.

The aortic diameter was significantly larger postoperative compared with preoperative at all aortic levels (Table 3 and Table 4 and Figure 2). At 40 mm proximal to the lowest renal artery baseline, which is above the level of the bare stent of the Zenith and Endurant endografts, the mean difference was 1.5 ± 1.8 mm (6.2 ± 7.3% increase compared with the preoperative diameter). At 10 mm distal to the lowest renal artery baseline, the mean difference was 2.7 ± 1.9 mm (12.6 ± 9.8% increase compared with the preoperative diameter). The diameter increase did not differ significantly between the different endograft types at all levels (*p* = 0.422, *p*= 0.331, *p* = 0.736, *p* = 0.217, and *p* = 0.289 for −40, −10, 0, 10, and 20 mm relative to the baseline), although this could be because of a type II error. 

The median planned oversizing of the pre-EVAR aortic neck diameter at baseline was 31% (22%, 40%); however, the effective post-EVAR oversizing was 20% (10%, 26%). As a result, 20 patients (27%) had an effective oversizing of <10%. Neck length was inversely correlated with planned oversizing (ρ = −0.376 [*p* = 0.001]). 

The three patients with a type 1A endoleak on the first post-EVAR CTA scan had a 0.4 mm (+1.3%), 1.5 mm (+5.4%), and 1.6 mm (+7.5%) diameter increase in the supra-renal aorta at 40 mm proximal to the baseline, which is comparable to other patients. At 10 mm distal to the baseline, however, these patients had 4.1 mm (+26%), 4.2 mm (+22%), and 4.8 mm (+32%) neck diameter increase. The Excluder, Zenith, and Endurant endografts were oversized by 46%, 39%, and 51%, resulting in effective oversizing of 23%, 29%, and 26%, respectively. The preoperative neck length of these patients was 16, 19, and 30 mm.

**Figure 3:**
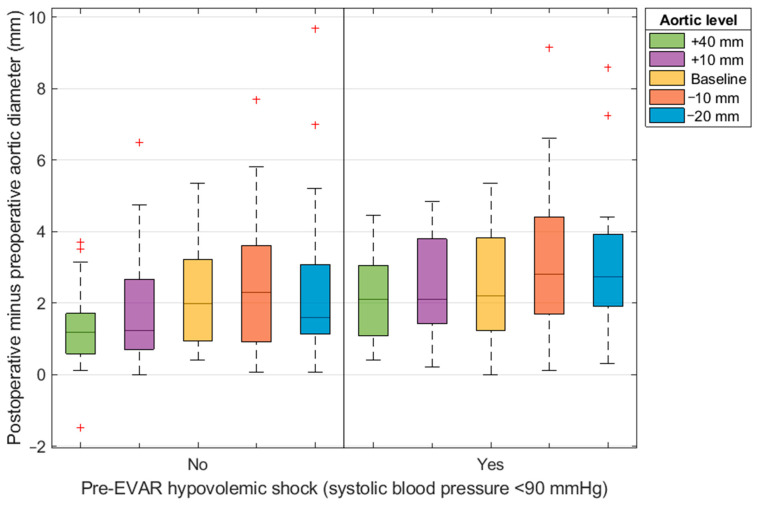
EVAR = endovascular aneurysm repair; diameter change at various levels of the aorta relative to the renal artery baseline for patients with and without preoperative hypovolemic shock (systolic blood pressure of <90 mmHg).

The aortic diameter increase at 40 mm proximal to the baseline correlated with the preoperative systolic blood pressure (R = −0.368 [*p* = 0.003]; Table 4). The supra-renal diameter increased 2.2 ± 1.4 mm in patients with hypovolemic-induced hypotension, and 1.3 ± 1.0 mm in patients without hypovolemic-induced hypotension. The diameter increase at 40 mm proximal to the renal artery baseline in patients with hypovolemic-induced hypotension is 69% higher than in patients without hypovolemic-induced hypotension (Figure 3). The difference in neck dilation between patients with and without hypovolemic shock was significant at the levels 40 and 10 mm above the lowest renal artery baseline (*p* = 0.005 and *p* = 0.038, respectively). The preoperative aortic diameter at baseline and the amount of planned oversizing did not correlate significantly with an aortic diameter increase (R = 0.077 [*p* = 0.515] and R = 0.058 [*p* = 0.622], respectively). The diameter change correlated significantly with the timing of the first postoperative CTA scan at the level 10 mm distal to the lowest renal artery baseline (R = 0.254, *p* = 0.029), but not at the supra-renal level 40 mm proximal to baseline (R = 0.004, *p* = 0.970). Patients with hypovolemic-induced hypotension had a slightly shorter time to the first post-operative CTA scan (34 ± 33 days) compared to patients without hypotension (41 ± 24 days); however, the difference was not significant (*p* = 0.309).

The median preoperative neck length was 18.6 (10.0, 28.3) mm. The median post-operative shortest apposition length of the endograft with the aortic wall was 13.9 (7.3, 27.2) mm. A median ratio of 0.7 (0.4, 1.2) of the preoperatively anticipated neck length was effectively used for apposition. Table 5 shows the correlation between the shortest apposition length and the shortest apposition length/neck length ratio with the diameter increase and the intended oversizing at 10 mm distal from the lowest renal artery baseline. Both parameters, the shortest apposition length and the ratio, correlated with neck diameter increase (ρ = −0.256 [*p* = 0.031] and ρ = −0.244 [*p* = 0.040]), but not with the intended oversizing (ρ = −0.157 [*p* = 0.192] and ρ = −0.183 [*p* = 0.126]). 

## 4. Discussion

In this retrospective series of patients with rAAA, a significant difference exists between pre- and postoperative supra- and infra-renal aortic diameters due to hypotension-induced aortic diameter decrease. This resulted in substantial aortic diameter increase post-EVAR which had a negative influence on the amount of circumferential apposition of the endograft in the aortic neck. These results are in line with previous reports in hypovolemic abdominal and thoracic aortic repair and porcine aortic models [7,13,14].

Insufficient oversizing or even undersizing increases the risk of a type IA endoleak, possibly affecting outcomes after ruptured EVAR. To achieve 10% to 20% effective oversizing, the endograft should be oversized by 30% to 40% compared with the pre-EVAR measured infra-renal neck. This is in accordance with the ESVS guidelines, which advises 30% oversizing for rAAA patients. This recommendation is, however, based on a single case study [4]. Too much effective oversizing (>30%) should be avoided as it has been associated with increased risk of endograft migration and AAA expansion, and may result in infolding of the endograft with associated type IA endoleaks. Previous studies also showed that the aortic neck of rAAA patients expand more and faster than elective patients. However, in the subgroup of ruptured patients, the median aortic neck diameter before EVAR was larger compared to the control group, which could have resulted in increased neck expansion [15]. The preoperative systolic blood pressure should therefore be taken into account when choosing the right amount of oversizing. Despite a slightly shorter time before the first postoperative CTA scan for patients with a systolic blood pressure of <90 mmHg, the average diameter increase in the supra-renal aorta was 69% larger than for patients with a systolic blood pressure of ≥90 mmHg. Besides measuring the intended oversizing on the preoperative CTA scan, the diameter should also be measured on the postoperative CTA scan to determine if the achieved effective oversizing is within the target range.

The 2.7 ± 1.9 mm infra-renal neck diameter increase in patients with rAAA was substantially more than the 0.9 ± 3.6 mm to 1.6 (1.0, 2.7) mm diameter increase reported for elective EVAR patients at the first postoperative CTA scan [11,16]. The neck diameter increase found in this study is comparable with >3 years of gradual aortic neck diameter increase in elective EVAR patients [17]. The amount of aortic diameter increase has an effect on the length of apposition that is achieved postoperative and on the ratio of the preoperative neck length that is used postoperatively for apposition of the endograft.

Previous studies reported that neck diameters can be measured in a vascular workstation with a precision of 0.0 to 0.5 mm, with 95% of the variance within 0.9 to 3.8 mm. Neck lengths can be measured with precision of 0.0 to 0.9 mm, and 95% of the variance within 2.0 to 4.1 mm [12,18]. In this study, it was attempted to minimize the measurement variability by having all measurements performed by one trained observer, thus eliminating inter-observer variability, according to a strict measurement protocol, and randomly checked by a second observer. The measurement variability probably accounts for the reduced postoperative diameter that is observed in a small proportion of the patients. 

This study has several limitations. Firstly, due to the retrospective study design, the data depended on what was reported in the patient files. The diastolic blood pressure was not documented for all patients, which is why only the systolic blood pressure could be included. Furthermore, the exact timing of the postoperative blood pressure measurements was not documented and could have varied between patients. Not all patients had their blood pressure measured just before the post-EVAR CT scan. A second limitation is the variety of implanted endografts. Each device exerts a different radial force onto the aorta which may be of influence on aortic dilatation. A third limitation is the absence of mid- and long-term follow-up for a substantial number of the patients. Many patients were referred from a different hospital and received further follow-up at the referring hospital. A second postoperative CTA scan was only available for 38 patients (42%), which is why a correlation with the risk of developing a type IA endoleak during follow-up is lacking. A fourth limitation is the result of a bias that is induced by only including patients who survived and underwent a postoperative CTA scan.

## 5. Conclusions

Hypovolemic-induced hypotension results in a significant decrease in the aortic diameter in patients presenting with an rAAA. This aortic diameter decrease in combination with the radial force of the endograft after EVAR may result in a substantial aortic neck diameter increase, and can cause insufficient oversizing and insufficient length of endograft apposition, which possibly increases the risk for a type IA endoleak. The amount of neck diameter increase correlates with preoperative hypotension. Future research should investigate the long-term clinical consequences of aortic diameter increase due to hypovolemic-induced hypotension.

## Figures and Tables

**Table 1:** Baseline total patient characteristics ^1^.

Variable	Value (%)
Age (years)	74 ± 7
Male sex	64 (87)
BMI (kg/m^2^)	26 ± 5
ASA Physical Status >II:	42 (58)
Hypertension [Systolic blood pressure > 140 mmHg]	39 (53)
Diabetes mellitus	11 (15)
Heart disease	7 (10)
COPD	13 (18)
Smoking:	
Current smoker	18 (25)
Former smoker	16 (22)
Never smoker	4 (5)
Unknown	36 (49)

^1^ Categorical data are presented as n (%); continuous data are presented as mean ± SD; ASA = American Society of Anesthesiologists physical status classification; BMI = body mass index; COPD = chronic obstructive pulmonary disease.

**Table 2:** Hemodynamic and renal parameters upon presentation at emergency department and post-EVAR.

Variable	Pre-EVAR (Mean ± SD)	Post-EVAR (Mean ± SD)	*p*-Value
Systolic blood pressure (mmHg)	106 ± 32	133 ± 24	<0.001
Heart rate (bpm)	81 ± 19	84 ± 22	0.497
eGFR (mL/min/1.73 m^2^)	57 ± 16	66 ± 21	0.002
Creatinine (µmol/L)	110 ± 33	95 ± 36	0.001

eGFR = estimated glomerular filtration rate. EVAR = endovascular aneurysm repair.

**Table 3:** Aortic neck diameters and oversizing measured on the preoperative and first postoperative computed tomography scans.

Level Relative to Lowest Renal Artery	PreOperative Diameter (mm)	Post-Operative Diameter (mm)	*p*-Value	Planned Pre-EVAR Oversizing (%)	Achieved Post-EVAR Oversizing (%)	*p*-Value
+40 mm	24.9 ± 2.7	26.3 ± 2.6	<0.001			
+10 mm	22.9 ± 2.8	24.9 ± 2.8	<0.001			
Baseline	22.0 ± 3.2	24.3 ± 3.1	<0.001	31 (22–40)	20 (10–26)	<0.001
−10 mm	22.7 ± 3.9	25.4 ± 3.7	<0.001	27 (19–36)	14 (7–23)	<0.001
−20 mm	24.4 ± 6.0	27.7 ± 6.3	<0.001	22 (11–28)	10 (1–16)	<0.001

EVAR = endovascular aneurysm repair.

**Table 4:** Correlation between preoperative systolic blood pressure and aortic diameter increase post-EVAR.

Level Relative to Lowest Renal Artery	Diameter Increase (mm)	Correlation with Systolic Blood Pressure (R)	*p*-Value
+40 mm	1.5 ± 1.8	−0.368	0.003
+10 mm	2.1 ± 1.5	−0.338	0.007
Baseline	2.2 ± 1.5	−0.204	0.108
−10 mm	2.7 ± 1.9	−0.387	0.002
−20 mm	3.4 ± 4.3	−0.115	0.371

EVAR = endovascular aneurysm repair.

**Table 5:** Spearman correlation between shortest apposition length and shortest apposition length/neck length ratio with neck diameter increase and the intended oversizing at 10 mm distal from the lowest renal artery baseline.

	Aortic Neck Diameter Increase		Intended Oversizing	
	Correlation (ρ)	*p*-Value	Correlation (ρ)	*p*-Value
Shortest apposition length	−0.256	0.031	−0.157	0.192
Shortest apposition length/neck length ratio	−0.244	0.040	−0.183	0.126

## Data Availability

Not applicable.
